# Motor Imagery EEG Classification for Patients with Amyotrophic Lateral Sclerosis Using Fractal Dimension and Fisher’s Criterion-Based Channel Selection

**DOI:** 10.3390/s17071557

**Published:** 2017-07-03

**Authors:** Yi-Hung Liu, Shiuan Huang, Yi-De Huang

**Affiliations:** 1 Graduate Institute of Mechatronic Engineering, National Taipei University of Technology, Taipei 10608, Taiwan; huangbl30815@gmail.com (S.H.); s0985321@gmail.com (Y.-D.H.); 2 Department of Mechanical Engineering, National Taipei University of Technology, Taipei 10608, Taiwan

**Keywords:** EEG, BCI, motor imagery, ALS, fractal dimension, channel selection, machine learning

## Abstract

Motor imagery is based on the volitional modulation of sensorimotor rhythms (SMRs); however, the sensorimotor processes in patients with amyotrophic lateral sclerosis (ALS) are impaired, leading to degenerated motor imagery ability. Thus, motor imagery classification in ALS patients has been considered challenging in the brain–computer interface (BCI) community. In this study, we address this critical issue by introducing the Grassberger–Procaccia and Higuchi’s methods to estimate the fractal dimensions (GPFD and HFD, respectively) of the electroencephalography (EEG) signals from ALS patients. Moreover, a Fisher’s criterion-based channel selection strategy is proposed to automatically determine the best patient-dependent channel configuration from 30 EEG recording sites. An EEG data collection paradigm is designed to collect the EEG signal of resting state and the imagination of three movements, including right hand grasping (RH), left hand grasping (LH), and left foot stepping (LF). Five late-stage ALS patients without receiving any SMR training participated in this study. Experimental results show that the proposed GPFD feature is not only superior to the previously-used SMR features (mu and beta band powers of EEG from sensorimotor cortex) but also better than HFD. The accuracies achieved by the SMR features are not satisfactory (all lower than 80%) in all binary classification tasks, including RH imagery vs. resting, LH imagery vs. resting, and LF imagery vs. resting. For the discrimination between RH imagery and resting, the average accuracies of GPFD in 30-channel (without channel selection) and top-five-channel configurations are 95.25% and 93.50%, respectively. When using only one channel (the best channel among the 30), a high accuracy of 91.00% can still be achieved by the GPFD feature and a linear discriminant analysis (LDA) classifier. The results also demonstrate that the proposed Fisher’s criterion-based channel selection is capable of removing a large amount of redundant and noisy EEG channels. The proposed GPFD feature extraction combined with the channel selection strategy can be used as the basis for further developing high-accuracy and high-usability motor imagery BCI systems from which the patients with ALS can really benefit.

## 1. Introduction

BCI enables individuals with severe motor disabilities to control devices or communicate with others by means of their brain activity. Brain activity can be measured by different modalities. Commonly used include EEG [1], functional magnetic resonance imaging (fMRI) [2], and near-infrared spectroscopy (NIRS) [3,4,5,6]. Among these, EEG is the most frequently used because of many advantages such as lower cost, better portability, and higher temporal resolution. Many EEG BCIs have been successfully tested on able-bodied subjects, yet patients with amyotrophic lateral sclerosis (ALS) who have really benefited from the BCI technologies are still relatively few [7]. One possible reason for the limited success of BCI on patients is the impairment in sensorimotor process [8], which may result in degenerated BCI performance [9]. Various BCIs for patients with ALS have been presented. The majority of these BCIs are based on P300 event-related potentials and slow cortical potentials (SCPs) (see [7,10] for a review). Only a few are focused on the sensorimotor rhythms (SMRs) induced by motor imagery [9,11,12,13]. Although P300 BCIs can provide higher information transfer rates, users need to gaze at target stimuli, which is infeasible for late-stage or completely locked-in ALS patients whose oculomotor control function has already been impaired [14]. Moreover, the control of SMRs by motor imagery can provide ALS patients with faster BCI communication than SCP [11]. Therefore, motor imagery is a more feasible approach for late-stage ALS patients to control a BCI.

Motor imagery is based on the modulation of SMRs which comprise μ (mu, 8–13 Hz) and β (beta, 13–30 Hz) rhythms from the sensorimotor area [15]. Changes of the SMRs, such as the contralateral side’s mu event-related desynchronization (ERD) and the ipsilateral side’s beta event-related synchronization (ERS) [16] induced by the imagination of unilateral hand movement, provides a BCI with discriminative EEG features to generate control commands [17,18,19]. However, such spatial-spectral EEG features may not be discriminative for patients with ALS due to the fact that the neurons in the motor cortex have been degenerated in ALS [20], leading to the impairment in the ability to modulate the SMRs [21,22]. Accordingly, although the motor imagery is more feasible for late-stage ALS patients to control BCIs, the mission of designing high-accuracy motor-imagery BCIs is very challenging [10,23].

### 1.1. Related Works

Previous studies have indicated the difficulties of achieving high motor imagery classification accuracy in ALS patients. In [11], four ALS patients’ classification accuracies in a motor imagery task (imagination of movement vs. resting) are 78% (CP3, right hand, 19.5–25.5 Hz), 81% (CP3, right hand, 17.5–20.5 Hz), 77% (CP4, left hand, 8.5–11.5 Hz), and 76% (Cz, feet, 18.5–24.5 Hz), respectively. These reported results are the average accuracies over the final three of 20 sessions, which means that the accuracy would be lower for patients without receiving any/much SMR training (i.e., motor imagery training). As also reported in [11], the average classification accuracy over the first three of the 20 sessions is near chance level (<60%) for the first patient. In [12], the classification accuracy of 80% is obtained from one ALS participant in a motor imagery task. The same motor imagery BCI is further tested on six patients, including three ALS patients without receiving SMR training and three patients with primary lateral sclerosis (PLS), using beta-band powers from 29 EEG channels as the features [13], where the average accuracy achieved by the patients is 83.3%. Recently, Geronimo et al. [9] test the classification accuracy of two different motor imagery tasks (left-hand grasping and right-hand grasping) by the use of band power features (5–30 Hz) extracted from six EEG channels (F3, F4, C3, C4, P3, P4). The average accuracy over the 25 ALS patients is merely 60%.

The results in these works [9,11,12,13] can give us several valuable directions to further develop more robust methods. First, all these works used more restrictive features (e.g., mu (8–13 Hz) and beta frequency band power features), mainly because multiple studies had previously demonstrated the frequency band and location of oscillatory changes due to motor imagery [24,25]. However, these restrictive features resulted in limited classification accuracy, as indicated in those studies involving ALS patients. Therefore, whether better classification performance can be achieved with utilization of other EEG features should be further investigated. Second, the EEG recording sites are located mainly at the region around the sensorimotor cortex, except in the study of [13] where the higher accuracy (83.3%) is based on the use of a high-density electrode montage (29 channels) covering not only the sensorimotor region but also other brain areas (frontal, parietal, temporal, and occipital). Furthermore, most fMRI studies have provided evidence of a spatial shift of function in motor area in patients with ALS [26]. All these findings suggest that the EEG signals from other brain regions may carry highly discriminable information during motor imagery. Finally, according to [11], the best EEG channel is patient-dependent. A method that can optimize the EEG channel configuration for each patient is required.

### 1.2. Proposed Work

The aims of the presented study is to investigate the possibilities of using fractal dimension as the feature to enhance the accuracy of motor imagery BCI for patients with ALS, and to develop an efficient method that can determine the optimal configuration of the EEG channels for each patient. Fractal dimension is a way to measure the complexity of a time series by estimating the self-similarity over some time interval [27]. Since the oscillatory brain activity varies with mental state, the fractal dimension (FD) can be used to characterize the complexity for EEG [28]. Its success has been shown in studies aiming to discriminate between patients (e.g., with major depression [29], stroke [30]) and healthy controls, and in the motor imagery EEG studies conducted on healthy subjects [31,32,33,34,35,36]. Among these works [31,32,33,34,35,36], two have compared the motor imagery classification performance between FD and SMR [31,36], and their reported results showed that FD was better than SMR in most cases. The study of [31] compared the FD estimated by Katz’s method with the band power (BP) features extracted from the EEG of C3, C4, and Cz on five able-bodied subjects. Their accuracy results based on an Adaboost classifier show that three subjects’ FDs achieved a lower error rate than BP, one subject’s FD and BP had the same accuracy, and the remaining one subject’s BP was better than FD. In [36], the average accuracy (85.50%) over three subjects of Higuchi’s FD was higher than that (81.30%) of BPs extracted from the same three channels (C3, Cz, and C4) using a Gaussian kernel-based support vector machine classifier. Therefore, FD might be also able to achieve better accuracy on ALS patients. To the best of our knowledge, the presented work is the first study that attempts to introduce the FD to the analysis on motor imagery EEG classification for patients with ALS. 

FD can be estimated by different methods, such as the detrended fluctuation analysis (DFA) method [37], Katz’s method [38], Higuchi method [39], rescaled range method [40], and the method by Grassberger and Procaccia (GP) [41]. Some previous works have compared these FD methods. The comparisons in [34] indicate that the Katz and Higuchi methods are comparable to each other, and are all better than the rescaled range method in terms of the motor imagery classification accuracy. The comparisons in [29] show that DFA and Higuchi have approximately the same accuracy, and the GP method outperforms the two FD methods (DFA and Higuchi) in terms of the EEG classification between depressed patients and controls. It seems that the GP method can achieve higher accuracy than the Higuchi method. However, those comparisons were based on motor-imagery EEG classification of able-bodied subjects [34] and the EEG discrimination between patients with major depressive disorder and controls [29]. No comparison has been made to indicate which is better in motor-imagery EEG classification for ALS patients. Therefore, we compare the two FD methods in the present study. We also compare the two kinds of fractal dimension features with the features that have been applied to the motor imagery classification in ALS, i.e., the SMR features. 

For the second aim, we apply Fisher’s criterion [42,43] to accomplish the task of channel selection for each patient. This method calculates the Fisher score of the fractal dimension feature computed from the EEG signal of each channel. The higher the value of the Fisher score is, the better the class separability of the channel’s EEG fractal dimension is. Based on the criterion, the optimal channel configuration for each patient can be determined automatically by selecting a smaller set of EEG channels with the highest Fisher scores. Moreover, BCI’s preparation stage is often time-consuming (applying electric-gel to the EEG electrodes, etc.). This drawback is further magnified if preparation is completed by people who are not the experts like the nursing staff in the hospital or the experimenters in the lab. ALS patients need assistance from their caregivers to set up the BCI. On the other hand, too many electrodes would also make the patients uncomfortable. According to the patients in our study and their family members, a BCI with at most five electrodes is preferred. Therefore, considering this factor of usability, we restrict our Fisher channel down-select to three options in this study, including 30, 5, and 1, where the 30-channel accuracy (i.e., without channel selection) is used as a baseline to examine the accuracy drop due to the channel reduction. Moreover, *K*-nearest neighbor (*K*-NN) and linear discriminant analysis (LDA) classifiers have been widely used in BCI studies [44,45,46]. In this study, we also compare the two based on the FD feature in different cases, including different tasks and different EEG configurations.

## 2. Materials and Methods

### 2.1. Participants

Five patients with ALS were recruited from the Taiwan Motor Neuron Disease Association and participated in this study. All gave informed consent. The ALS functional rating scale-revised (ALSFRS-R) [47] was recorded for each patient. The ALSFRS-R questionnaire assesses the bulbar, limb, and respiratory functions, and quantifies the overall physical function by a total score from 0 (worst) to 48 (normal). The stages of the patients were also evaluated based on the staging criteria for ALS [48,49]. The stages of ALS are defined as the following: stage 1 (symptom onset), stage 2A (diagnosis), stage 2B (involvement of second region), stage 3 (involvement of third region), stage 4A (need for gastrostomy), and stage 4B (need for non-invasive ventilation). A patient can belong to stage 4A and 4B simultaneously. The patients belonging to stage 4A and/or 4B can be considered as late-stage ALS. All patients’ data are listed in Table 1. Prior to this study, the patients did not receive any training related to the regulation of the brain rhythms including the SMR training by motor imagery.

### 2.2. EEG Recording and Setting

**Figure 1:**
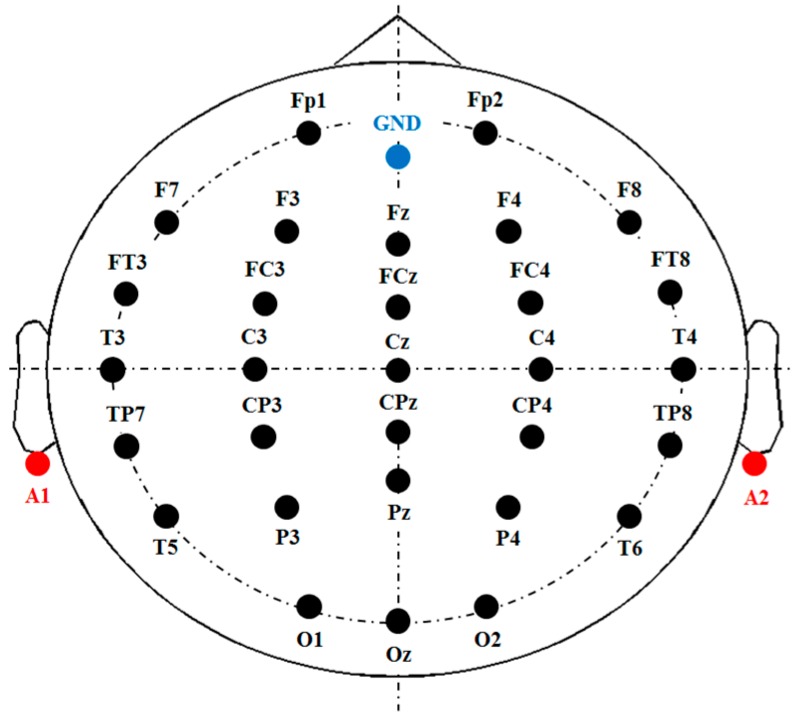
Electrode placement. Thirty-three channels are used, among which two are references (A1, A2), one is the ground (GND), and the remaining are used for EEG recording. The positions of the electrodes follow the international 10–20 system.

EEG signals were recorded by a 33-channel electro-cap. The positions of the Ag/AgCl electrodes followed the international 10–20 system (see Figure 1). Electrode gel was applied to the electrodes to keep the impedance below 10 K Ohm. The ground channel was at the forehead, and the reference channels were at the mastoids (A1 and A2). The remaining 30 channels were all used to record EEG signals for analysis. Electrooculography (EOG) signals were recorded by the electrodes attached at the right side of the right eye and above of the left eye, respectively. Both EEG and EOG were amplified, band-pass filtered (0.5–100 Hz), and converted to digital signals with a sampling rate of 500 Hz using the NuAmp amplifier produced by NeuroScan Inc. The ocular artifacts were then removed from the EEG signals using the artifact removal software tool provided by the NeuroScan. The processed EEG signals were all stored in a personal computer for further offline analysis.

### 2.3. Preparation

The first four patients sat in a wheelchair (patients Sub1 and Sub3) or normal armchair (Sub2 and Sub4) while facing a 20-inch computer screen. The patient Sub5 reclined in the bed. We projected the computer screen on the wall synchronously by a projector so that the instructions presented during the experiment could be clearly perceived by the patient Sub5.

**Figure 2:**
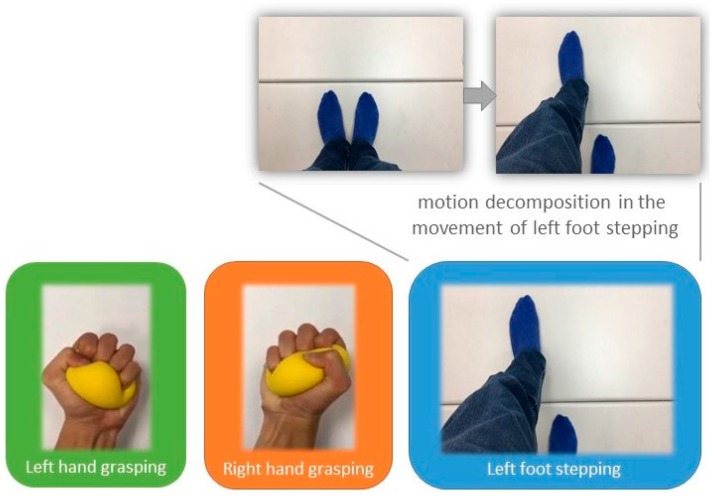
The motor imagery tasks include left hand grasping, right hand grasping, and stepping across a black line in front of the patient with left foot. The three pictures were presented during the preparation stage so that the patients could practice the designated motor imagery following the movements shown in the pictures.

Three kinds of motor imagery tasks were designed in this study to test the classification accuracy of each motor imagery and no imagery (i.e., resting), including grasping an object by left hand (LH), grasping an object by right hand (RH), and stepping across a line in front of the patient with left foot (LF). All patients were provided with three pictures showing the three kinds of movements, respectively (Figure 2). They were asked to practice the imagination of the motions during the preparation stage (the stage of applying electro-gel to the 30 electrodes). They were also informed of the motion decomposition in each movement in order to ensure the motor imagery contents across the patients were consistent. For example, the movement of left foot stepping was decomposed to two motions: standing then stepping across the line with the left foot, as shown in Figure 2.

### 2.4. EEG Data Collection

**Figure 3:**
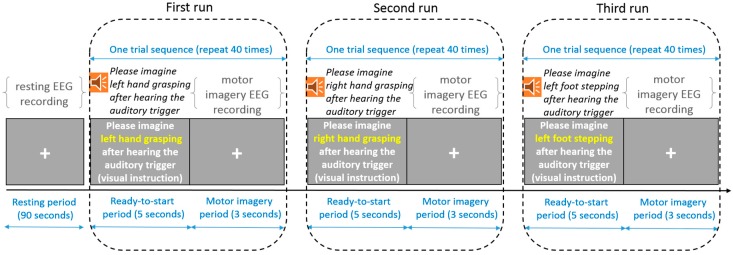
Resting and motor imagery EEG data collection. The motor imagery data collection consisted of three runs separated by 1- to 3-min breaks, and each run had 40 trials. One trial started with a 5-s ready-start period followed by a 3-s motor imagery period during which the participants performed the designated motor imagery task according to both visual and auditory cues presented in the ready-to-start period.

The motor-imagery EEG data collection session consisted of three runs separated by 1- to 3-min breaks, each run had 40 motor-imagery trials. The motor imagery tasks to be performed in the three runs are LH, RH, and LF, respectively. Prior to the session, there was a resting period of 90 s during which the patients were asked to keep relaxed, try to gaze at the cross centered at the screen and try not to think anything on purpose (Figure 3). The resting EEG signals of 90 s were segmented into 40 epochs of 3-s length, and there was a 1-s overlap between two consecutive EEG epochs.

Each trial consisted of a 5-s ready-to-start period and a 3-s motor imagery period. During the ready-to-start period, both visual and auditory cues were presented to the patients. Taking the first run as an example, the auditory cue was the sentence: please imagine left hand grasping after hearing an auditory trigger. Its text content was also shown on the screen as a visual cue. In the beginning of the motor imagery period, there was an auditory trigger. The patients started to imagine the designated movement after they perceived this auditory cue, and they were asked to gaze at the fixation cross on the screen while imaging the movement. The trials repeated 40 times in each run. Thus, the motor imagery EEG collection session lasted to about 20 min (8 s×40 trials×3 runs+2 min×2 breaks between runs). For each patient participant, forty EEG signals of 3 s were obtained for each task (resting, LH imagery, RH imagery, LF imagery).

### 2.5. Feature Extraction

#### 2.5.1. GPFD

The GP algorithm estimates the correlation dimension (D_2_) or FD of an attractor in phase-space domain. Let $\bar{x}=\left[x\left(1\right),\;x\left(2\right),\;\ldots ,\;x\left(N\right)\right]$ denote the EEG signal of 3 s, where *N* is the length of the signal (N=500 Hz×3 s=1500). The EEG signal can be reconstructed as a set of *M*-dimensional vectors as

(1) $$y\left(i\right)=\left[\bar{x}\left(i\right),\;\bar{x}\left(i+\tau \right),\;\bar{x}\left(i+2\tau \right),\ldots ,\;\bar{x}\left(i+\left(M-1\right)\tau \right)\right],\;i=1,2,\ldots ,\;N-\left(M-1\right)\tau$$

where τ is the time delay and M is the embedding dimension. The time delay is a free parameter which needs to be determined experimentally. The correlation integral

(2) $$C\left(r\right)=\frac{2}{N\left(N-1\right)}\overset{N-\left(M-1\right)\tau }{\underset{\begin{array}{c} i,j=1\\ i\neq j \end{array}}{\sum }}H\left(r-\left|y\left(i\right)-y\left(j\right)\right|\right)$$

stands for the probability that the set of the distance between two different reconstructed vectors y(i) and y(j) (i.e., |y(i)−y(j)|, ∀i≠j) fall into the cell of size r at a given embedding dimension M, where *H* is a Heaviside function defined as H(y)=1 if y>0;H(y)=0 otherwise. The correlation dimension $d_{c}$ is given by

(3) $$d_{c}\left(M\right)=\underset{r\to 0}{\lim}\frac{\log C\left(r\right)}{\log r}$$

The slope of logC(r) versus logr at a given M can be estimated over the region where logC(r) is approximately linear in logr by linear least-squares fitting. The slope of the line is the $d_{c}$ value at the given M. Moreover, the value of $d_{c}$ gradually increases with increase of M, and then achieves saturation. The saturation value is defined as the FD [29]. However, in numerical computation, the saturation value would not maintain at a constant, but varies slightly as the M value further increases. Accordingly, the FD was determined based on the steps as follows,

*Step 1*. initialize M=2;
*Step 2*. M=M+1;
*Step 3*. calculate the $d_{c}$ value at the given M;
*Step 4*. repeat step 2 and step 3 until $\left|d_{c}\left(M\right)-d_{c}\left(M-1\right)\right|<\epsilon$;
*Step 5*. $\mathrm{FD}=d_{c}\left(M\right)$.

where ϵ is a positive number (we set ϵ=0.001). An excessively large value of epsilon could result in an *M* value for which the corresponding $d_{c}$ value has not yet saturated (e.g., *M* < 10). On the contrary, an excessively small value of epsilon could lead to the problem that no *M* value can be obtained. In this study, the ϵ value was decided by trial and error. By using the setting of ϵ=0.001, the FDs of all EEG signals were obtained, and their *M* values were within the range of *M* > 10.

**Figure 4:**
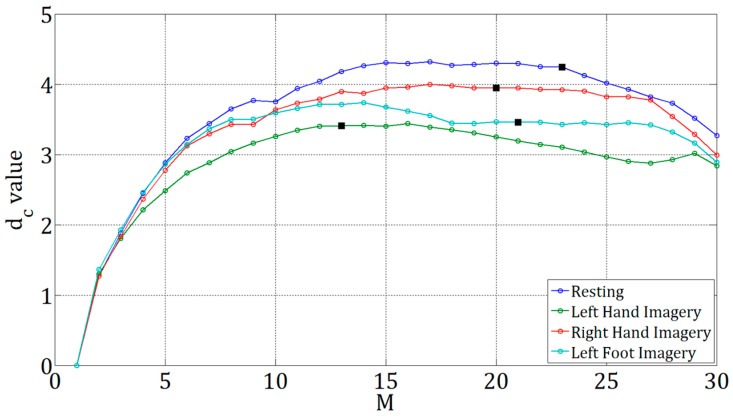
Plots of $d_{c}$ against *M* for four 3-s EEG signals recorded under resting, left hand imagery, right hand imagery, and left foot imagery, respectively, and were all recorded at F7 from the patient participant Sub1. The correlation dimension $d_{c}$ value marked in black in each curve is the FD estimated by the GP method. In this example, the $d_{c}$ values for resting, left hand, right hand, and left foot conditions reached the plateau before M=30 (i.e., M = 23, 20, 21, and 13, respectively). The FDs of the four signals are 4.2479, 3.4062, 3.9438, and 3.4577, respectively. The time delay was set as 50 for the four cases.

Examples of the $d_{c}-M$ plots are shown in Figure 4, where each curve was calculated based on a 3-s EEG signal of a mental task. The four curves were based on the signals of resting, LH imagery, RH imagery, and LF imagery, respectively (all from patient Sub1 and recorded at F7). It can be observed from Figure 4 that the M values corresponding to the FDs (the $d_{c}$ values marked in black square) for the four different tasks are different (M = 23, 20, 21, 13 for the four tasks). According to our experiment, the GPFDs of all the EEG signals were found within the range of M=10 to M=30. Also, the *M* values associated with the GPFDs for different trials and different participants were not necessarily the same, depending the result by the aforementioned steps for the FD calculation. 

#### 2.5.2. HFD

The Higuchi method estimates the FD of a time series directly in the time domain without reconstructing the strange attractor, and gives a reasonable FD estimate for a short-time segment [32,39]. For an EEG signal of *N* samples：$\bar{y}=\left[y\left(1\right),\;y\left(2\right),\;\ldots ,\;y\left(N\right)\right]$, *k* new signals are reconstructed by

(4) $$y_{m}^{k}=\left[y\left(m\right),y\left(m+k\right),y\left(m+2k\right),\ldots ,\;y\left(m+int\left(\frac{N-m}{k}\right)k\right)\right],\;m=1,2,\ldots ,k$$

where the integers m and k stand for the initial time point and the time interval, respectively, and int(·) denotes the integer function. The length of each curve $y_{m}^{k}$ is computed as

(5) $$L_{m}\left(k\right)=\frac{1}{k}\left[\frac{N-1}{int\left(\frac{N-m}{k}\right)k}\left(\overset{int\left(\frac{N-m}{k}\right)}{\underset{i=1}{\sum }}\left|y\left(m+ik\right)-y\left(m+\left(i-1\right)k\right)\right|\right)\right]$$

The length $L_{m}\left(k\right)$ represents the normalized sum of absolute values of differences between two consecutive points of distance k. The length of the curve for each time interval k, is computed as the average of the m curves:

(6) $$L\left(k\right)=\frac{1}{k}\overset{k}{\underset{m=1}{\sum }}L_{m}\left(k\right)$$

**Figure 5:**
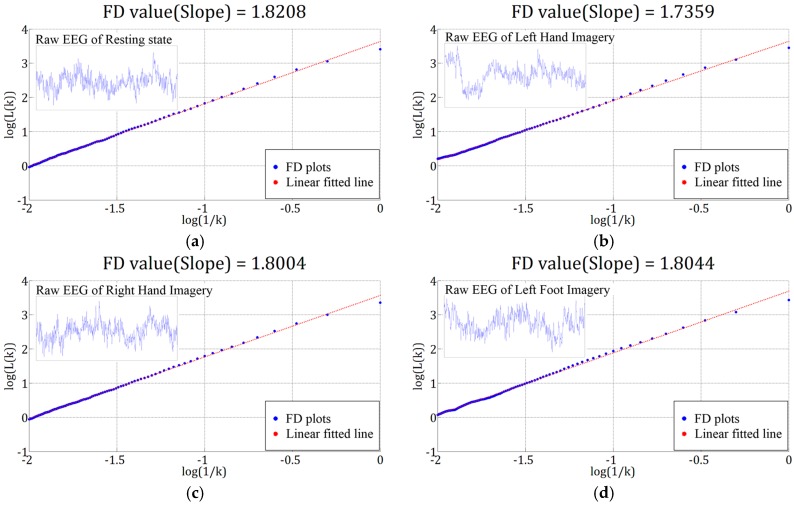
Log-Log plots of L(k)  versus 1/k based on the 3-s EEG signals from (**a**) resting, (**b**) left hand (LH) imagery, (**c**) right hand (RH) imagery, and (**d**) left foot (LF) imagery, respectively. The four signals were recorded at the same channel F7, and were collected from the patient participant Sub1. In this example, the fractal dimensions (FDs) estimated by the Higuchi method for the four tasks are 1.8202, 1.7359, 1.8004, and 1.8004, respectively.

The signal has a fractal dimension of FD if

(7) $$L\left(k\right)\propto k^{FD}$$

for different values of k. The FD value can be obtained by applying the least-squares linear fitting to calculate the slope of log(L(k)) against log(1/k). The slope of the line is the FD of the EEG signal. The maximum k value, $k_{max}$ was set as 100 in this study. The FD value ranges from 1 (deterministic) to 2 (stochastic signal). Thus, the higher the value of the FD is, the higher the complexity of the signal is. Figure 5 shows the example of log-log plots of L(k) versus 1/k of four different mental tasks.

### 2.6. Channel Selection Based on Fisher’s Criterion

The aim of channel selection is to select the most discriminative EEG channels from the original 30 channels by applying the Fisher’s criterion. The FD features from the 30 channels are concatenated to a feature vector (called data hereafter) of dimension q, where q=30. Let $x_{ij}\in R^{q}$ denotes the *j*th training data of class i, where i=1,…, c, $m_{i}$ is the mean of the training data of the *i*th class, and m is the mean of the training data from all classes. In this study, we focused on the classification problem in a motor imagery task (resting vs. motor imagery). Thus, c=2. The within-class $S_{w}$ and the between-class $S_{b}$ scatter matrices are calculated by

(8) $$S_{w}=\overset{c}{\underset{i=1}{\sum }}P_{i}\left(\frac{1}{n_{i}}\overset{n_{i}}{\underset{j=1}{\sum }}\left(x_{ij}-m_{i}\right){\left(x_{ij}-m_{i}\right)}^{t}\right)$$

and

(9) $$S_{w}=\overset{c}{\underset{i=1}{\sum }}P_{i}\left(\frac{1}{n_{i}}\overset{n_{i}}{\underset{j=1}{\sum }}\left(x_{ij}-m_{i}\right){\left(x_{ij}-m_{i}\right)}^{t}\right)$$

respectively, where $n_{i}$ denotes the number of data in the *i*th class, the superscript *‘t’* stands for the transpose of a matrix, and $P_{i}=n_{i}/\overset{c}{\underset{j=1}{\sum }}n_{j}$ is the prior probability of the *i*th class. The class separability for the *f*th feature (f=1,…,q) can be represented by the Fisher score F(f) as

(10) $$F\left(f\right)=\frac{S_{b}\left(f\right)}{S_{w}\left(f\right)},\;f=1,2,\ldots ,q$$

where $S_{b}\left(f\right)$ and $S_{w}\left(f\right)$ are the *f*th diagonal elements of $S_{b}$ and $S_{w}$, respectively. The higher the value of F(f) is, the better the class separability of the feature extracted from the *f*th channel is. Based on the class separability criterion, we can select d top channels among the 30 ones. The d features from the selected channels are concatenated to a feature vector. 

### 2.7. Classification and Parameter Optimization

Both *K*-NN and linear discriminant analysis (LDA) classifiers were used for testing the two-class classification accuracies of RH imagery vs. resting (RH vs. R), LH imagery vs. resting (LH vs. R), and LF imagery vs. resting (LF vs. R). The classification accuracy was computed by leave-one-out cross validation (LOO-CV). For each two-class classification problem, there are totally 80 EEG data (40 from resting, and 40 from a motor imagery). One of the 80 data is used for testing, and the remaining ones were used for training the classifier. This procedure repeated until every data served as the test data once. 

**Figure 6:**
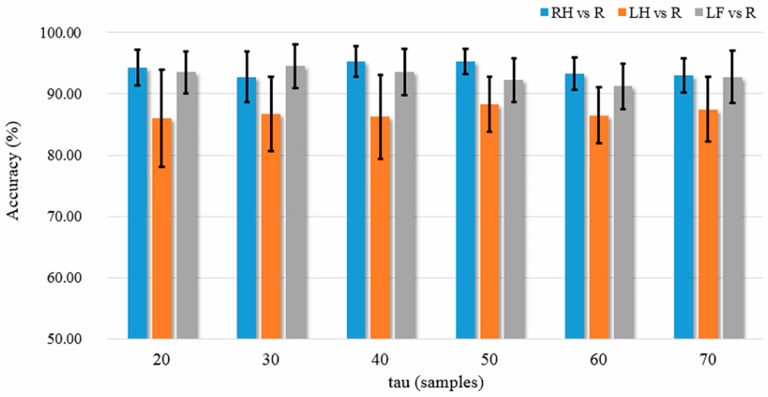
Cross validation accuracies based on 30 channels versus different values of time delay. The error bars are the standard error of the mean.

Parameters of the methods were optimized by the cross validation procedure combined with grid searching method. In this study, only the GPFD method involves free parameter, i.e., the time delay τ (in sample points). We searched for the optimum value of time delay in the set τ={20, 30, 40, 50, 60, 70}; namely there were 6 parameter grids. The LOO-CV 30-channel classification accuracies for different time delay values and tasks are shown in Figure 6. It can be observed from Figure 6 that the best accuracies for both RH-R and LH-R classification tasks occurred at τ=50. The best τ value for LF-R classification (94.50%) was 30, which was only slightly higher than the accuracy (92.25%) of τ=50. Therefore, we set τ=50 for all classification tasks. The results reported in the following are the highest LOO-CV classification accuracies. 

## 3. Results and Discussion

### 3.1. Results

**Figure 7:**
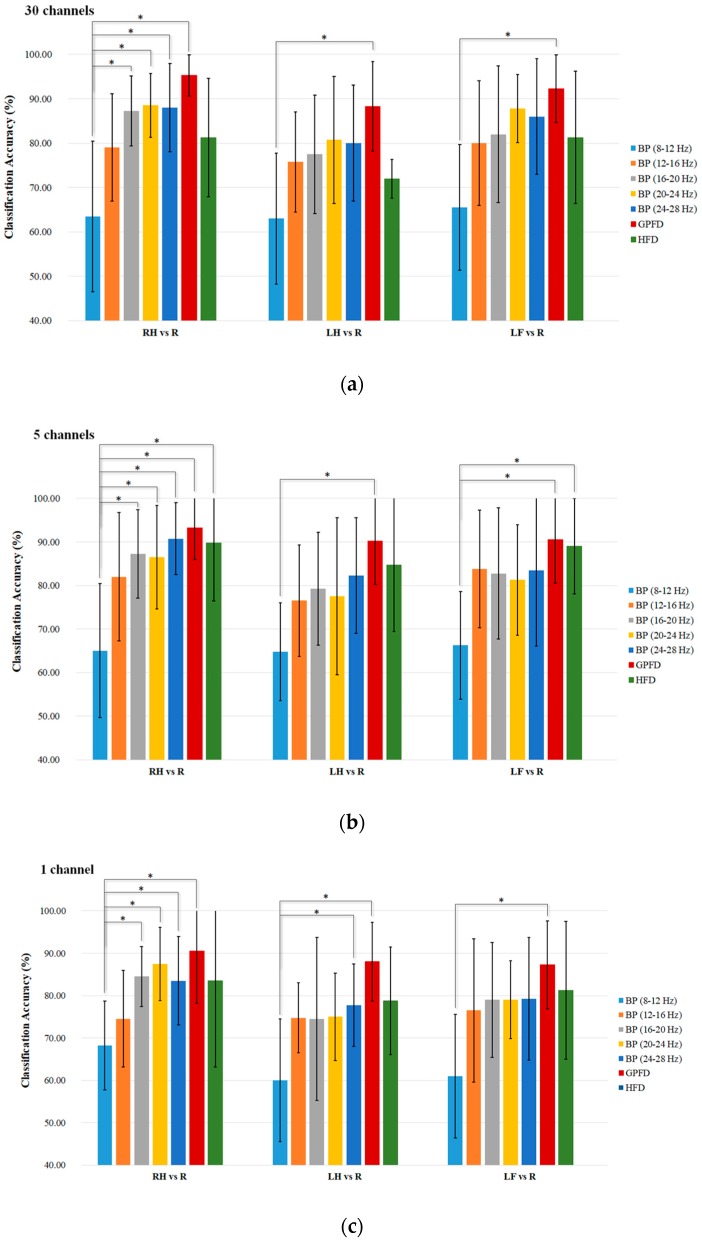
Comparison of the average *K-*NN classification accuracies among different feature extraction methods using (**a**) 30 EEG channels, (**b**) 5 EEG channels, and (**c**) single EEG channel. The five channels are the ones have the top five Fisher scores among the 30, and the channel used in (**c**) is the best among the five in (**b**). * indicates significant difference between methods at *p* < 0.05.

The average *K*-NN classification accuracies over the five patient participants among different feature extraction methods are listed in Figure 7a–c, where the results shown in the three subplots are based on all 30 EEG channels (without channel selection), the top five channels, and the best channel, respectively. We used the Wilcoxon rank sum test to statistically examine the difference of classification accuracy between different feature extraction methods, because the one-sample Kolmogorov Smirnov test rejected the null hypothesis of normal distribution of the data (*p* < 0.05). Table 2, Table 3 and Table 4 list the top five channels and the corresponding Fisher values in RH-R, LH-R, and LF-R classification conditions. Different subbands in beta band may have different levels of beta rebound induced by a motor imagery [25,50]. Therefore, we divided the mu and beta bands into five subbands, and computed the BP of the five subbands for the purpose of comparison.

Among the BP features, the mu band performs the worst (only 60–70%) for all tasks and channel configurations. Overall, the high beta band of 20–28 Hz performs the best. Taking the RH-R classification task as an example, the BP of 24–28 Hz achieves high accuracy of 88% in 30-channel configuration (Figure 7a), and accuracy of 90.75% in the configuration with five optimal channels (Figure 7b). When using one channel, the BP of 20–24 Hz can still give high RH-R classification accuracy of 87.5%. In contrast, the GPFD method provides a higher accuracy than all the BPs and HFD, and the averaged classification accuracy of GPFD is significantly higher than that of the mu-band BP in all tasks and all channel configurations (*p* < 0.05). Also, GPFD is still capable of maintaining a high accuracy even if the number of channels is largely reduced from 30 to 5, and even from 5 to 1. Taking RH-R classification as an example, the accuracies achieved by GPFD in 30, 5, and 1 channel configurations are 95.25%, 93.25%, and 90.50%, respectively. The performance drop is very limited, and the accuracy remains to be above 90% even if only one single channel (i.e., top channel) is used (Figure 7c).

**Figure 8:**
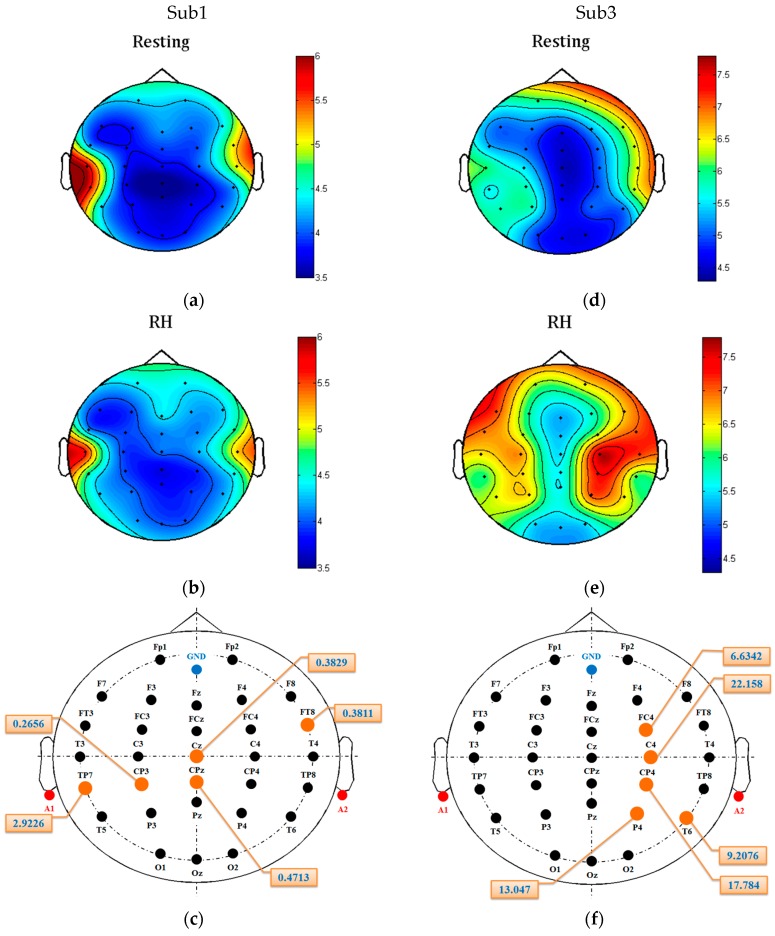
Average GPFD topoplots, where (**a**,**b**) display the resting and right hand (RH) imagery topoplots of patient Sub1, respectively, (**c**) shows the top five channels and their Fisher scores, (**d**,**e**) display the resting and RH imagery topoplots of patient Sub3, respectively, and (**f**) shows the patient Sub3’s top five channels and their Fisher scores. The values shown in (**a**,**b,d,e**) are average GPFD values. The top five channels in (**c**,**f**) are marked in yellow. The value of each best channel is the Fisher score. For example, the Fisher score of the patient Sub1’s channel Cz is 0.3829. The best channels for patients Sub1 and Sub3 are TP7 and C4, respectively.

The above results have shown that the top five channels are able to achieve high accuracy. However, it is unclear whether the channels with the smallest Fisher scores are really noisy. Therefore, we further conducted an experiment to examine whether the Fisher’s criterion-based channel selection can remove noisy/redundant channels. Table 5 shows the comparison of average *K*-NN classification accuracies between the top-five-channel (listed in Table 2, Table 3 and Table 4) and the worst-five-channel configurations, where the worst five EEG channels are the ones having the lowest Fisher score values among the 30 channels. The results show that most accuracies of the worst-five-channel configurations are close to chance level, and the accuracies of the top five channels are much higher. Taking RH-R classification as an example, the BP of 20–24 Hz only gives accuracy of 62.50% by the worst-five-channel configuration, while its accuracy achieves 86.50% when using the top five channels. Similarly, the difference of RH-R classification accuracy between the two configurations achieves almost 30% (93.25% − 65.50% = 27.75%) when the GPFD feature was used. The comparisons indicate that some brain regions are close to useless while some are very helpful for the discrimination between resting and motor imagery. Figure 8 shows two patients’ GPFD topoplots of resting and RH imagery states. For patient Sub1, no significant changes can be observed in some areas, such as frontal and occipital. For patient Sub3, significant difference between the two states is observed from the area around C4, while some other regions such as occipital and the right prefrontal (FP2) show almost no difference. Figure 8c and f show that the top-five-channel configurations for the two patients are different even if the features used are the same (i.e., GPFD). The above results and observations demonstrate that (1) channel selection is necessary, (2) best EEG channel configuration is patient-dependent, and (3) the best patient-dependent five-channel configuration obtained by the Fisher’s criterion is able to achieve high classification accuracy.

The results listed in Figure 7 have shown that the GPFD method is superior to other methods. However, the classification accuracies in Figure 7 are based on the simple classifier *K*-NN. We conducted another experiment to see if the GPFD-based motor imagery classification performance in ALS can be improved by using the more sophisticated classifier LDA. According to Table 6, LDA performs better than *K*-NN in most cases. However, the improvement is very limited. The maximum improvement is only 2.25% (from 88.25% of *K*-NN to 90.50% of LDA), occurring in the LH-R classification using 30 channels. One possible reason is that when a simple *K*-NN classifier is used, the resulting classification accuracy based on GPFD feature is already very high (88.25%). Therefore, the space of the accuracy that can be improved by other more advanced classifiers like LDA is limited (only 100% − 88.25% = 11.75%), which might explain why LDA outperforms *K*-NN by only 2.5%. However, the error reduction rate is high, because 11.75% − 9.50%/11.75% = 21.28%. From the error-reduction point of view, LDA improved a lot.

### 3.2. Discussion

**Figure 9:**
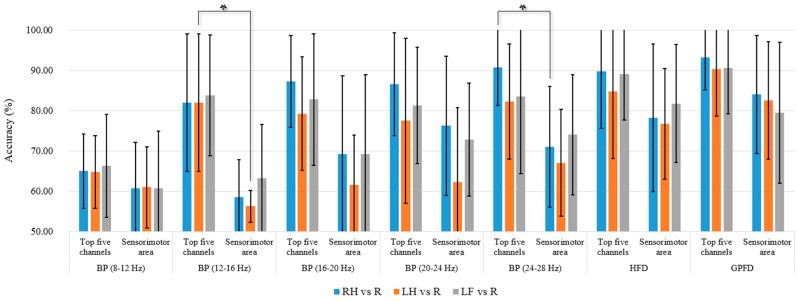
Comparison of the accuracies between the top-five-channel configuration and the five channels (C3, Cz, C4, CP3, CP4) within the sensorimotor area. * indicates significant difference between EEG configurations (top five channels and the five channels on the sensorimotor area) for each method at *p* < 0.05.

No previous studies have been focused on the FD-based motor imagery classification for patients with ALS. Nevertheless, our results have shown the success of the use of FD (particularly the GPFD) as the feature for classification. As mentioned in Section 1, the previous works on motor imagery classification studies involving ALS patients ([9,11]) were all based on the SMR feature, i.e., the mu and beta BPs of EEG signals recorded at the sensorimotor area. To compare with the previous approach, we calculated the features (BPs of five subbands within the alpha and beta bands, GPFD, HFD) of the EEG signals from five sensorimotor channels (C3, Cz, C4, CP3, CP4). The comparison of accuracies between the sensorimotor area and the top-five-channel configuration (listed in Table 2, Table 3 and Table 4) is shown in Figure 9, where *K*-NN is used as the classifier, and the accuracies in the top five channels are the same as those in Figure 7b. According to Figure 9, it is clear that the BPs from the sensorimotor area do not provide satisfactory average classification accuracy (<80% in all cases). Our results are consistent with the ones of earlier study [9] and the latest study [11], where the accuracies are all below 80%. In [9], the five patients’ classification accuracies of hand motor imagery vs. resting are inside of the range of 70–80%, and the accuracy is lower than 60% for a patient without receiving SMR training. In [11], the average accuracy is around 60%. The results of our study and the previous studies [9,11] show that the BPs from the sensorimotor area are not robust features for the motor imagery classification in ALS. On the other hand, our RH-R and LF-R classification results show that the classification accuracy of high beta BPs (20–24 Hz and 24–28 Hz) from the sensorimotor area is higher than 70%, which is also much higher than the accuracies of around 60% reported in [9,11] in which the EEG features are also the BPs from the sensorimotor area. Such difference could be due to the fact that the classification in our study was processed offline while these studies [9,11] calculated classification using online feedbacks of motor imagery. Since offline analysis operates in a synchronous (cue-based) mode, the offline accuracy is often higher than the accuracy of online testing (i.e., asynchronous or self-paced mode).

The results in Figure 9 also reveal that the top-five-channel configuration is more robust than the five channels within the sensorimotor area. For all features (BP, GPFD, HFD) and all classification tasks (RH-R, LH-R, LF-R), the top-five-channel configuration achieves a better average classification accuracy than the five channels within the sensorimotor area. For the BP feature of 12–16 Hz, the LH-R classification accuracy of top five channels is significantly higher than the accuracy of the five sensorimotor channels (*p* < 0.05). For the BP feature of 24–28 Hz, the top-five-channel configuration also achieves a significantly higher accuracy in RH-R classification than the five sensorimotor channels (*p* < 0.05). Moreover, we can see from Table 2, Table 3 and Table 4 that most of top five channels do not fall within the sensorimotor area. Taking the patient Sub1 as an example (BP of 20–24 Hz, RH-R classification), the patient’s top five channels (TP7, T4, FP2, T3, O2) does not contain any from the sensorimotor area. All these comparison results suggest that the searching of best EEG recording sites for ALS patients should not be restricted within the sensorimotor area.

In this study, we have compared the performances between two FD methods (GPFD and HFD) with BPs of different subbands. The mean classification accuracies by the two FD methods were all different for all tasks, which was due to the fact that the two methods estimate the FD by different approaches. Moreover, GPFD achieved higher mean accuracies than HFD for all classification tasks and all channel configurations. One possible reason is that Higuchi’s method is very sensitive to noise [51]. Such high noise sensitivity could limit the discriminating ability of the FD features estimated by Higuchi’s method, because in this study the inputs were signal-trial raw EG signals which had low signal-to-noise ratio.

We have compared the GPFD and FD with BP features of different subbands. In BCI studies, common spatial pattern (CSP) [19,50,52,53] and autoregressive model (AR) [54] have been also widely used. CSP transforms a filtered multi-channel EEG E ($N_{c}\times N$, where $N_{c}$ denotes the number of channels) to new EEG signal $E_{N}$ ($N_{c}\times N$) by a transform W: $E_{N}=WE$, where the signal E needs to be spectrally filtered in advance [52]. The training phase of CSP is to obtain the matrix W (see the steps summarized in [50]). The normalized log powers of the first *p* and the last *p* rows of the new signal matrix $E_{N}$ are optimal for the discrimination between two classes. Therefore, a CSP feature vector consists of 2*p* features, where *p* is a free parameter of CSP method. AR is a time-domain feature extraction method which involves only one parameter, the order of the model. Given an order value, the ARM finds the best fit to the real EEG signal using the least-square method. Supposing that the order is set as d, then for each channel’s EEG signal there are d AR coefficients extracted. The AR coefficients from each of the $N_{c}$ channels are concatenated to a feature vector of $N_{c}\times d$ dimension. The searching values for d include 2, 3, 4, 5, 6, and 7. In our experiment, the optimal CSP parameter and the AM parameter were all determined by the LOO-CV procedure determined in Section 2.7. According to Figure 7, GPFD is superior to other features. Therefore, here we only compared the GPFD with CSP and AR features, and the accuracies of other features (BP and HFD) are already shown in Figure 7. The classification results based on *K*-NN classifier are listed in Table 7. According to Table 7, the best RH-R classification accuracy in the three channel configurations (30, top five, and top one) are 95.25%, 93.25%, and 90.50%, respectively, which are all obtained by GPFD. However, beta-CSP, i.e., raw EEG signals were band-pass filtered by a 3-order Butterworth filter (13–30 Hz) and then sent into CSP for feature extraction, performs better than mu-CSP in two conditions (30 and top five channels). Also, beta-CSP is better than GPFD for LH-R classification when 30 channel were used. Overall, CSP and GPFD are comparable to each other in the condition of 30 channels. However, the results show that GPFD was much better than CSP in top-five-channel condition, especially for LF-R classification, where GPFD achieved a high accuracy of 90.50% while the accuracies of both mu-CSP and beta-CSP were all below 80%. On the other hand, CSP is based on the decomposition of the signal parameterized by a matrix that projects the signals in the original recording space to the ones in the surrogate sensor space. However, this decomposition relies on at least two EEG channels; otherwise the spatial covariance matrix of CSP cannot be decomposed (see [55] for detailed formulation). In other words, at least two channels are required to record EEG signals when using the CSP method, which is its limitation in use. Therefore, the CSP method cannot be applied in top-one-channel condition, as shown in Table 7 where N/A mean “not available”. This is one advantage of GPFD over CSP, and GPFD can still achieve high accuracy (90.50% in RH-R classification) even if only one channel was used. AR and mu-CSP gives similar accuracies. However, beta-CSP outperforms AR greatly. The comparison results listed in Table 7 indicate that GPFD is more suitable in motor imagery classification for ALS patients, especially when there are only few channels used for EEG recording. Although the classifier design is beyond the scope of our study, it is expected that the classification accuracy can be further improved by using advanced classifiers, the convolutional neural networks (CNN) [56] for example. 

The successful implementation of GPFD feature extraction and Fisher’s criterion-based channel selection strategy has several implications for further development of motor imagery BCI systems for patients with ALS. First, the current study tested the accuracies for three different classification tasks (RH vs. R, LH vs. R, and LF vs. R). Each of the binary classifications can be the basis for further developing a two-state asynchronous BCI [57] by which the ALS patients can respond to their family members’ questions (express “yes” for example) by performing a motor imagery, which can increase their quality of life. Such simple function is useful for late-stage ALS patients whose speaking function are severely impaired (for example the patient Sub5 in this study), and is extremely useful for ALS patients who are in the completely locked-in state. Currently, our methods were tested on the EEGs from the late-stage ALS patients who are still able to communicate (but very slow). It is unknown whether it is possible to successfully transfer the methods (including the EEG data collection paradigm) to completely locked-in ALS patients. However, a recent study has evidenced that cognitively intact patients ALS patients produced motor imagery decoding accuracies better than cognitively impaired patients [9]. Moreover, in our data collection stage (Figure 3), a voice instruction is presented before every motor imagery period. Thus, the locked-in patients may be able to follow the instruction to perform the instructed motor imagery tasks even if they are unable to watch the computer screen. Therefore, it is believed that there exists such possibility as long as their cognition process is still normal (can hear, and follow the voice instructions). Nevertheless, it should be further justified in the future. Second, this study introduced the Fisher’s separability measure to automatically select a set of discriminating channels which are patient-dependent. This can be applied to effectively and efficiently calibrate the best EEG recording sites for different users/patients before training the motor imagery classification model. Although the results have demonstrated that the best channel configuration is capable of achieving high accuracy, the accuracy is not necessarily optimal in practice. In other words, the best channel configuration is not necessarily the best. The main reason is that the Fisher’s separability criterion calculates the Fisher scores of the features (or channels) separately, and does not consider the joint class separability of multiple features [42]. In other words, some features that do not have relatively high Fisher scores may provide high or even higher classification accuracy when these features are used together as the input to the classifier. To deal with this drawback, some advanced methods could be applied, such as the joint mutual information [58] and the evolutionary computation algorithms like particle swarm optimization (PSO) [59]. Last, our results have shown that high accuracy can be achieved by the use of low-density electrodes (5 or 1) with GPFD feature. However, the results of the methods were based on a commercialized EEG amplifier, which is very expensive. To improve the motor imagery BCI’s applicability, there is a need to develop a low-cost and portable EEG acquisition and preprocessing module so that the BCI system based on the proposed methods can really be used to help patients with ALS.

## 4. Conclusions

Motor imagery BCI has shown success on able-bodied individuals. Yet, there is still limited efficacy in late-stage ALS, which may be due to the fact that typical features, spectral information in the mu and beta bands, may not be as discriminative for ALS patients. To deal with this problem, we have proposed in this paper a method by combining the GPFD feature and Fisher criterion-based channel selection strategy. Although the two separate methods have already been used in the BCI community, the use of GPFD and its combination with channel selection is novel for ALS patients’ motor imagery classification. Our results have demonstrated that the proposed use of GPFD is superior to other features that have been used in previous studies involving ALS patients, and is able to achieve high accuracy (~90%) even when there is only one channel (the one selected by the proposed automatic subject-independent channel selection strategy). In summary, based on the results, it can be concluded that the contribution of the proposed method is three-fold. First, the use of the GPFD feature can provide motor imagery BCIs for ALS patients with higher accuracy. Second, the combination of the GPFD feature and the Fisher’s criterion-based channel selection strategy is able to automatically determine the best patient-dependent channel configuration from 30 EEG recording sites. Based on only a few selected channels, the GPFD can still maintain high average accuracy, which greatly improves the usability of the BCI for ALS patients. Finally, our analysis results suggested that most of the discriminating channels for ALS motor imagery classification do not fall on the sensorimotor area. To our knowledge, this is a new finding in the EEG-based BCI community, and also supports the fMRI evidence that there is a spatial shift of function in the motor area in ALS patients [18].

## Figures and Tables

**Table 1:** Patient description.

Patient	Age	Sex	TSSO ^1^	ALSFRS-R ^2^	Limb Function	Speech	Stage
Sub1	59	M	11 y	16	No	Yes	4A & 4B
Sub2	65	M	7 y	7	Minimal	Slow	4A & 4B
Sub3	64	M	20 mo	12	Minimal	Yes	4B
Sub4	39	F	44 mo ^3^	24	Minimal	Slow	4A
Sub5	47	M	11 y	5	No	Slow	4A & 4B

^1^ TSSO: time since symptom onset, ^2^ ALSFRS-R: ALS functional rating scale-revised, ^3^ mo: month.

**Table 2:** Top five channels and the corresponding Fisher values for each feature in RH vs. R condition.

sub	8–12 Hz BP	12–16 Hz BP	16–20 Hz BP	20–24 Hz BP	24–28 Hz BP	GPFD	HFD
1	T5 (0.1959)	TP7 (0.4776)	TP7 (0.8866)	TP7 (0.6420)	TP7 (1.0184)	TP7 (2.9226)	TP7 (2.1797)
	CP3 (0.1868)	T3 (0.1518)	T4 (0.7818)	T4 (0.3990)	T4 (0.7183)	CPz (0.4713)	FT8 (0.6036)
	T3 (0.1326)	Pz (0.1195)	FP2 (0.3299)	FP2 (0.3753)	FP1 (0.2776)	Cz (0.3829)	CPz (0.2593)
	O2 (0.1236)	FT7 (0.1073)	O2 (0.1559)	T3 (0.1249)	O2 (0.2209)	FT8 (0.3811)	O1 (0.2634)
	TP7 (0.1117)	O2 (0.0767)	T3 (0.1158)	O2 (0.1170)	FP2 (0.1556)	CP3 (0.2656)	Pz (0.1515)
2	Pz (0.1162)	T6 (0.5264)	FCz (0.4075)	F7 (0.3662)	FT8 (0.4431)	TP8 (0.5208)	O2 (0.1715)
	P3 (0.1137)	FC4 (0.4914)	FC4 (0.3178)	FP1 (0.3457)	C4 (0.2878)	T3 (0.3017)	Oz (0.1489)
	CP3 (0.0775)	F4 (0.2750)	F7 (0.2691)	FCz (0.3146)	F8 (0.2746)	FCz (0.2071)	FP1 (0.1249)
	CPz (0.0696)	FC3 (0.1727)	F4 (0.2173)	Fz (0.1943)	FT7 (0.2606)	C3 (0.2057)	O1 (0.1245)
	P4 (0.0657)	Cz (0.1598)	FC3 (0.2071)	F3 (0.1711)	T4 (0.2472)	FT7 (0.1684)	T5 (0.0864)
3	FT8 (0.1150)	T4 (1.1110)	T4 (1.0073)	T4 (1.8131)	T4 (1.4962)	C4 (22.1579)	C4 (9.5388)
	Pz (0.1117)	TP8 (0.6196)	TP8 (0.9739)	TP8 (1.4694)	TP8 (1.1764)	CP4(17.7842)	P4 (6.5059)
	Oz (0.0868)	T3 (0.4998)	T3 (0.7371)	F3 (1.1074)	FC3 (1.0906)	P4 (13.0471)	CP4 (5.9313)
	F8 (0.0713)	TP7 (0.2949)	CP3 (0.7054)	T3 (0.9613)	TP7 (1.0091)	T6 (9.2076)	T6 (4.1303)
	F7 (0.0495)	FP1 (0.2803)	FP1 (0.6020)	CP4 (0.8266)	F3 (0.9516)	FC4 (6.6343)	FC4 (3.3493)
4	FT7 (0.1673)	F8 (0.1450)	TP7 (0.3527)	F7 (1.3163)	F8 (0.8605)	T5 (2.3012)	T5 (0.7612)
	T3 (0.0570)	Oz (0.1393)	F8 (0.2183)	F8 (0.4023)	F7 (0.1334)	FC4 (1.0024)	FC4 (0.5805)
	CP3 (0.0554)	F7 (0.1398)	O1 (0.1501)	O1 (0.3579)	O1 (0.0926)	Pz, (0.8049)	P3 (0.5124)
	C3 (0.0505)	O1 (0.1380)	F7 (0.1210)	Oz (0.3201)	TP7 (0.0856)	P3 (0.7944)	Oz (0.5050)
	TP7 (0.0458)	O2 (0.1350)	CP3 (0.1209)	TP7 (0.3143)	Oz (0.0824)	Fz (0.7880)	Pz (0.4517)
5	FP2 (1.0668)	FP2 (1.4845)	FP2 (1.5629)	FP2 (1.4995)	T4 (1.4758)	TP8 (4.0383)	TP8 (2.5587)
	FP1 (0.9840)	TP8 (0.9154)	TP8 (1.2318)	F7 (1.4014)	FP2 (1.4747)	T4 (2.5665)	P4 (2.1619)
	FT7 (0.8580)	FP1 (0.7829)	FP1 (1.1796)	TP8 (1.1532)	FP1 (1.3258)	P4 (2.4334)	T4, (1.9509)
	F8 (0.8472)	F8 (0.3947)	T4 (1.1297)	FP1 (1.0076)	FT7 (1.3033)	C4 (1.6472)	C4 (1.7615)
	F3 (0.8374)	F7 (0.3658)	CP4 (0.9431)	FT7 (0.8936)	F7 (1.2617)	CP4 (1.3791)	C3 (1.5695)

**Table 3:** Top five channels and the corresponding Fisher values for each feature in LH vs. R condition.

sub	8–12 Hz BP	12–16 Hz BP	16–20 Hz BP	20–24 Hz BP	24–28 Hz BP	GPFD	HFD
**1**	TP7 (0.1351)	TP7 (0.2906)	TP7 (0.6980)	TP7 (0.3016)	FT8 (0.3469)	TP7 (1.3889)	TP7 (2.1183)
	TP8 (0.0670)	FT8 (0.1265)	TF8 (0.1432)	FT8 (0.1337)	TP7 (0.1313)	CPz (0.6460)	P3 (0.8449)
	P4 (0.0611)	FT7 (0.0981)	T4 (0.0754)	FT7 (0.0902)	FP7 (0.0779)	O1 (0.5262)	CPz (0.7107)
	O2 (0.0500)	T5 (0.0529)	O2 (0.0454)	T4 (0.0673)	T4 (0.0747)	P3 (0.5262)	CP3 (0.4847)
	T4 (0.0467)	T3 (0.0526)	FT7 (0.0333)	T5 (0.0536)	T5 (0.0607)	T5 (0.5088)	Pz (0.3650)
**2**	P3 (0.1425)	FC3 (0.1658)	FC3 (0.1317)	FC3 (0.1825)	FC3 (0.4775)	TP8 (0.7588)	TP8 (0.2160)
	Pz (0.1316)	C3 (0.1503)	C3 (0.1289)	F7 (0.1088)	FC4 (0.2909)	T6 (0.2034)	FP1 (0.0863)
	CP3 (0.0870)	T6 (0.1500)	T6 (0.1282)	C3 (0.0737)	F8 (0.2580)	FT8 (0.1806)	P3 (0.0840)
	T6 (0.0866)	TP8 (0.1451)	F4 (0.1156)	FT7 (0.696)	F4 (0.2351)	F8 (0.1679)	O1 (0.0815)
	O2 (0.0418)	F4 (0.1300)	FC4 (0.1100)	FCz (0.0657)	FT8 (0.2008)	F4 (0.1531)	T5 (0.0803)
**3**	Pz (0.0785)	T4 (0.8316)	T4 (1.4278)	C4 (1.5888)	T4 (1.4773)	CP3 (7.5453)	P3 (1.5629)
	CPz (0.0390)	T3 (0.5088)	C4 (0.8031)	T4 (1.1867)	F7 (0.7968)	P3 (7.0727)	TP8 (1.2314)
	Oz (0.0335)	F7 (0.1977)	F7 (0.1428)	TP8 (0.1362)	TP8 (0.2109)	T5 (4.5642)	CP3 (0.9180)
	Cz (0.0297)	FP1 (0.1459)	TP8 (0.1123)	CP4 (0.1117)	CP4 (0.1894)	FT8 (2.6046)	F7 (0.5414)
	T4 (0.0235)	TP8 (0.1381)	CP4 (0.0776)	F7 (0.0907)	C3 (0.1807)	T3 (1.9923)	O1 (0.5411)
**4**	FT7 (0.0542)	FT8 (0.2108)	T5 (0.0796)	T5 (0.2058)	T5 (0.2268)	Fz (0.4379)	FC4 (0.2708)
	T3 (0.0472)	T4 (0.1761)	O1 (0.0793)	P3 (0.1353)	Fz (0.1363)	FC4 (0.3620)	Pz (0.2141)
	T5 (0.0353)	Oz (0.1710)	FT8 (0.0515)	Pz (0.1061)	FP1 (0.0745)	Cz (0.3444)	CPz (0.1951)
	P3 (0.0339)	O1 (0.1487)	Oz (0.0492)	CPz (0.0985)	CP3 (0.0745)	FCz (0.3265)	P3 (0.1744)
	CP3 (0.0297)	F7 (0.1100)	F8 (0.0412)	Oz (0.0649)	F7 (0.0534)	Oz (0.2655)	CP3 (0.1717)
**5**	FP2 (0.7118)	FP2 (1.0015)	FP2 (0.9555)	CP3 (1.2064)	F7 (2.2860)	T4 (3.8428)	T4 (2.2796)
	FP1 (0.6990)	FP1 (0.7181)	F3 (0.8529)	C3 (1.0922)	FT7 (1.4914)	TP8 (2.7892)	TP8 (2.0638)
	F7 (0.6279)	T3 (0.4920)	FP1 (0.8094)	FP2 (1.0032)	FP1 (1.3404)	P4 (1.9385)	C3, (1.8043)
	F8 (0.5995)	TP8 (0.4740)	T4 (0.8026)	TP8 (0.9364)	T4 (1.2849)	C4 (1.8107)	C4 (1.6101)
	F3 (0.5892)	FT7 (0.2995)	C3 (0.7651)	FC3 (0.8981)	C3 (1.0573)	CP4 (1.2856)	P4 (1.6054)

**Table 4:** Top five channels and the corresponding Fisher values for each feature in LF vs. R condition.

sub	8–12 Hz BP	12–16 Hz BP	16–20 Hz BP	20–24 Hz BP	24–28 Hz BP	GPFD	HFD
**1**	TP8 (0.0983)	T4 (0.5434)	T4 (0.7272)	T4 (0.7545)	T4 (0.8082)	CP4 (2.7924)	TP7 (2.8712)
	P4 (0.0652)	T3 (0.3338)	F8 (0.6317)	CP3 (0.4574)	T3 (0.6576)	TP7 (1.7434)	CP4 (2.4801)
	T4 (0.0433)	TP7 (0.2698)	TP7 (0.4421)	T3 (0.4488)	TP7 (0.3488)	C4 (1.5566)	T4 (1.6187)
	C3 (0.0363)	C4 (0.0824)	FC4 (0.3896)	T6 (0.4156)	FT7 (0.2678)	FC4 (1.3140)	C4 (1.4083)
	TP7 (0.0355)	FT7 (0.0397)	Fz (0.3703)	FC3 (0.3544)	C4 (0.2244)	T4 (1.2547)	P4 (1.3327)
**2**	Pz (0.2704)	T6(0.3515)	F4 (0.4160)	FP2 (0.3385)	T3 (0.5032)	FT8 (0.5611)	TP8 (0.1155)
	P3 (0.2291)	T3(0.2691)	F7 (0.3449)	FP1 (0.3038)	FP2 (0.4180)	T3 (0.1328)	T5 (0.0859)
	CPz (0.1975)	TP8 (0.2269)	T6 (0.3429)	TP8 (0.2709)	FT7 (0.3611)	T5 (0.1255)	O1 (0.0787)
	CP3 (0.1885)	F4 (0.2152)	FC4 (0.3394)	Cz (0.2687)	T6 (0.3563)	FP2 (0.878)	P3 (0.0784)
	P4 (0.0657)	O2 (0.2068)	FP2 (0.3149)	FCz (0.0657)	F4 (0.3550)	TP7 (0.0669)	P4 (0.0756)
**3**	T4 (0.2269)	F7 (2.7868)	F7 (2.8897)	F7 (1.5145)	F7 (2.1683)	C4 (25.2850)	C4 (8.3864)
	TP8 (0.0641)	TP8 (0.2692)	T4 (1.3730)	C4 (1.4156)	C3 (1.6969)	CP4(20.6432)	CP4 (5.1367)
	F7 (0.0634)	TP7 (0.2405)	C4 (0.8541)	T4 (1.3621)	FC3 (1.4402)	FC3(13.8415)	FC4 (4.9778)
	FT8 (0.0419)	T4 (0.2150)	C3 (0.8371)	C3 (1.3085)	C4 (1.2559)	C3 (11.2478)	P4 (4.9573)
	FP1 (0.0378)	F3 (0.1817)	FC3 (0.6426)	FT8 (1.2335)	T4 (1.2525)	F3 (10.6492)	FC3 (4.7763)
**4**	P3 (0.0664)	Cz (0.0809)	CP3 (0.2001)	Pz (0.3259)	Cz (0.0998)	FCz (0.9769)	Fz (0.4666)
	CP3 (0.0646)	FCz (0.0619)	Cz (0.1675)	CPz (0.3119)	Pz (0.0996)	Fz (0.6215)	Oz (0.3230)
	T5 (0.0599)	Fz (0.0484)	P3 (0.1618)	P4 (0.2125)	P3 (0.0971)	Pz, (0.4877)	CPz (0.2814)
	C3 (0.0595)	FT7 (0.0461)	CPz (0.1575)	O1 (0.2026)	CPz (0.0958)	FC4 (0.4635)	Pz (0.2349)
	O1 (0.0465)	F8 (0.0436)	Pz (0.1416)	Oz (0.1926)	O1 (0.0922)	TP7 (0.4519)	TP7 (0.2339)
**5**	FP2 (1.0830)	FP2 (0.6187)	FP1 (1.2658)	FT7 (0.9768)	FT7 (1.1204)	P4 (1.8536)	C3 (1.4460)
	FP1 (1.0581)	T3 (0.4771)	F3 (1.0748)	T4 (0.6783)	F7 (0.5620)	T3 (1.0703)	T3 (1.3716)
	F7 (1.0344)	FP1 (0.4640)	F7 (1.0565)	F3 (0.5796)	T4 (0.4857)	FT8 (0.9700)	T4 (1.3633)
	FT7 (1.0052)	F8 (0.4436)	FT7 (0.8939)	F7 (0.5622)	T3 (0.4718)	TP7 (0.8080)	FC3 (1.3311)
	F8 (1.0001)	F7 (0.3675)	T4 (0.8543)	T3 (0.4731)	F3 (0.4100)	FT7 (0.7736)	TP7 (1.2434)

**Table 5:** Comparison of average *K-*NN classification accuracies between the best-five-channel and the worst-five-channel configurations in different features and tasks (in %). Here, “Ave.” means average over the subjects.

Sub	Features	Top Five Channels			Worst Five Channels		
		RH vs. R	LH vs. R	LF vs. R	RH vs. R	LH vs. R	LF vs. R
1	BP (8–12 Hz)	61.25	68.75	56.25	55.00	53.75	57.50
	BP (12–16 Hz)	93.75	80.00	92.50	35.00	60.00	53.75
	BP (16–20 Hz)	93.75	83.75	87.50	58.75	51.25	56.25
	BP (20–24 Hz)	82.50	81.25	85.00	52.50	47.50	63.75
	BP (24–28 Hz)	92.50	78.75	90.00	47.50	57.50	56.25
	GPFD	98.75	97.50	97.50	65.00	58.75	62.50
	HFD	97.50	97.50	97.50	55.00	52.50	56.25
2	BP (8–12 Hz)	65.00	57.50	66.25	50.00	51.25	57.50
	BP (12–16 Hz)	66.25	60.00	78.7	56.25	43.75	45.00
	BP (16–20 Hz)	70.00	61.25	80.00	56.25	52.50	73.75
	BP (20–24 Hz)	66.25	57.50	62.50	80.00	51.25	67.50
	BP (24–28 Hz)	75.00	65.00	82.50	50.00	56.25	62.50
	GPFD	81.25	77.50	72.50	60.00	48.75	60.00
	HFD	66.25	75.00	77.50	60.00	63.75	68.75
3	BP (8–12 Hz)	63.75	60.00	65.00	55.00	52.50	56.25
	BP (12–16 Hz)	90.00	86.25	97.50	47.50	50.00	48.75
	BP (16–20 Hz)	95.00	86.25	98.75	45.00	52.50	67.50
	BP (20–24 Hz)	97.50	96.25	93.75	48.75	41.25	53.75
	BP (24–28 Hz)	97.50	97.50	100.00	51.25	48.75	55.00
	GPFD	100.00	100.00	100.00	81.25	52.50	96.25
	HFD	100.00	95.00	100.00	76.25	55.00	93.75
4	BP (8–12 Hz)	55.00	58.75	56.25	37.50	51.25	42.50
	BP (12–16 Hz)	61.25	68.75	60.00	46.25	48.75	40.00
	BP (16–20 Hz)	81.25	68.75	56.25	53.75	58.75	51.25
	BP (20–24 Hz)	90.00	55.00	70.00	65.00	68.75	63.75
	BP (24–28 Hz)	90.00	73.75	51.25	60.00	67.50	53.75
	GPFD	88.75	77.50	86.25	61.25	60.00	56.25
	HFD	86.25	60.00	76.25	61.25	48.75	47.50
5	BP (8–12 Hz)	80.00	78.75	87.50	71.25	68.75	76.25
	BP (12–16 Hz)	98.75	87.50	90.00	47.50	52.50	55.00
	BP (16–20 Hz)	96.25	96.25	91.25	70.00	72.50	82.50
	BP (20–24 Hz)	96.25	97.50	95.00	66.25	77.50	71.25
	BP (24–28 Hz)	98.75	96.25	93.75	66.25	81.25	78.75
	GPFD	97.50	98.75	96.25	60.00	78.75	53.75
	HFD	98.75	96.25	93.75	77.50	87.50	71.25
Ave.	BP (8–12 Hz)	65.00	64.75	66.25	53.75	55.50	58.00
	BP (12–16 Hz)	82.00	76.50	83.75	46.50	51.00	48.50
	BP (16–20 Hz)	87.25	79.25	82.75	56.75	57.50	66.25
	BP (20–24 Hz)	86.50	77.50	81.25	62.50	57.25	64.00
	BP (24–28 Hz)	90.75	82.25	83.50	55.00	62.25	61.25
	GPFD	93.25	90.25	90.50	65.50	59.75	65.75
	HFD	89.75	84.75	89.00	66.00	61.50	67.50

**Table 6:** *K*-NN and LDA classification results based on GPFD feature (in %).

Channels (num.)	*K*-NN Classifier			LDA Classifier		
	RH vs. R	LH vs. R	LF vs. R	RH vs. R	LH vs. R	LF vs. R
All (30)	95.25	88.25	92.25	95.25	90.50	93.50
Top five (5)	93.25	90.25	90.50	93.50	88.75	91.75
The best (1)	90.50	88.00	87.25	91.00	88.50	88.50

**Table 7:** Comparison between GPFD, AR, and CSP features using *K-*NN classifier (in %).

Methods	30 Channels			Top Five Channels			Top One Channel		
	RH-R	LH-R	LF-R	RH-R	LH-R	LF-R	RH-R	LH-R	LF-R
GPFD	95.25	88.25	92.25	93.25	90.25	90.50	90.50	88.00	87.25
AR	75.75	74.50	74.75	65.25	69.75	71.50	72.50	70.25	69.00
CSP (mu)	83.00	75.75	79.50	64.25	62.00	72.50	N/A	N/A	N/A
CSP (beta)	92.25	90.25	91.50	88.75	86.00	78.00	N/A	N/A	N/A

## References

[1] Lotte F., Congedo M., Lécuyer A., Lamarche F., Arnaldi B. (2007). A review of classification algorithms for EEG-based brain–computer interfaces. J. Neural Eng..

[2] Porro C.A., Francescato M.P., Cettolo V., Diamond M.E., Baraldi P., Zuiani C., Bazzocchi M., diPrampero P.E. (1996). Primary motor and sensory cortex activation during motor performance and motor imagery: A functional magnetic resonance imaging study. J. Neurosci..

[3] Khan M.J., Hong M.J., Hong K.-S. (2014). Decoding of four movement directions using hybrid NIRS-EEG brain-computer interface. Front. Hum. Neurosci..

[4] Naseer N., Hong M.J., Hong K.-S. (2014). Online binary decision decoding using functional near-infrared spectroscopy for development of a brain-computer interface. Exp. Brain Res..

[5] Naseer N., Hong K.-S. (2015). fNIRS-based brain-computer interfaces: A review. Front. Hum. Neurosci..

[6] Liu X., Hong K.-S. (2017). Detection of primary RGB colors projected on a screen using fNIRS. J. Innov. Opt. Health Sci..

[7] Marchetti M., Priftis K. (2015). Brain–computer interfaces in amyotrophic lateral sclerosis: A metanalysis. Clin. Neurophysiol..

[8] Strong M.J., Grace G.M., Freedman M., Lomen-Hoerth C., Woolley S., Goldstein L.H., Murphy J., Shoesmith C., Rosenfeld J., Leigh P.N. (2009). Consensus criteria for the diagnosis of frontotemporal cognitive and behavioural syndromes in amyotrophic lateral sclerosis. Amyotroph. Later. Scler..

[9] Geronimo A., Simmons Z., Schiff S.J. (2016). Performance predictors of brain–computer interfaces in patients with amyotrophic lateral sclerosis. J. Neural Eng..

[10] Moghimi S., Kushki A., Guerguerian A.M., Chau T. (2012). A review of EEG-based brain-computer interfaces as access pathways for individuals with severe disabilities. Assist. Technol..

[11] Kübler A.K., Nijboer F., Mellingerp J., Vaughan T.M., Pawelzik H., Schalk G., McFarland D.J., Birbaumer N., Wolpaw J.R. (2005). Patients with ALS can use sensorimotor rhythms to operate a brain-computer interface. Neurology.

[12] Bai O., Lin P., Vorbach S., Floeter M., Hattori N., Hallett M. (2008). A high performance sensorimotor beta rhythm-based brain–computer interface associated with human natural motor behavior. J. Neural Eng..

[13] Bai O., Lin P., Huang D., Fei D., Floeter M. (2010). Towards a user friendly brain-computer interface: Initial tests in als and pls patients. Clin. Neurophysiol..

[14] Jacobs L., Bozian D., Heffner R.R., Barron S.A. (1981). An eye movement disorder in amyotrophic lateral sclerosis. Neurology.

[15] Pfurtscheller G., Neuper C. (2001). Motor imagery and direct brain-computer communication. Proc. IEEE.

[16] Pfurtscheller G. (2001). Functional brain imaging based on ERD/ERS. Vis. Res..

[17] Liao K., Xiao R., Conzalez J., Ding L. (2014). Decoding individuals finger movements from one hand using human EEG signals. PLoS ONE.

[18] Yong X., Menon C. (2015). EEG classification of different imaginary movements with the same limb. PLoS ONE.

[19] Yang B., Li H., Wang Q., Zhang Y. (2016). Subject-based feature extraction by using fisher WPD-CSP in brain-computer interfaces. Comput. Methods Progr. Biomed..

[20] Nihei K., McKee A.C., Kowall N.W. (1993). Patterns of neuronal degeneration in the motor cortex of amyotrophic lateral sclerosis patients. Acta Neuropathol..

[21] Santhosh J., Bhatia M., Sahu S., Anand S. (2004). Quantitative EEG analysis for assessment to ‘plan’ a task in amyotrophic lateral sclerosis patients: A study of executive functions (planning) in ALS patients. Cogn. Brain Res..

[22] Kasahara T., Terasaki K., Ogawa Y., Ushiba J., Aramaki H., Masakado Y. (2012). The correlation between motor impairments and event-related desynchronization during motor imagery in ALS patients. BMC Neurosci..

[23] Hohmann M.R., Fomina T., Jayaram V., Widmann N., Förster C., Müller V., Hagen J., Synofzik M., Schölkopf B., Schöls L. A cognitive brain-computer interface for patients with amyotrophic lateral sclerosis. Proceedings of the International Joint Conference on IEEE Systems, Man, and Cybernetics.

[24] Pfurtscheller G., Lopes da Silva F.H. (1999). Event-related EEG/MEG synchronization and desynchronization: Basic principles. Clin. Neurophysiol..

[25] Hashimoto Y., Ushiba J. (2013). EEG-based classification of imaginary left and right foot movements using beta rebound. Clin. Neurophysiol..

[26] Lulé D., Ludolph A.C., Kassubek J. (2009). MRI-based functional neuroimaging in ALS: An update. Amyotrop. Lateral Scler..

[27] Mandelbrot B.B. (1982). The Fractal Geometry of Nature.

[28] Preissl H., Lutzenberger W., Pulvermüller F., Birbaumer N. (1997). Fractal dimensions of short EEG time series in humans. Neurosci. Lett..

[29] Hosseinifard B., Moradi M.H., Rostami R. (2013). Classifying depression patients and normal subjects using machine learning techniques and nonlinear features from EEG signal. Comput. Methods Progr. Biomed..

[30] Zappasodi F., Olejarczyk E., Marzetti L., Assenza G., Pizzella V., Tecchio F. (2014). Fractal dimension of EEG activity senses neuronal impairment in acute stroke. PLoS ONE.

[31] Boostani R., Moradi M.H. (2004). A new approach in the BCI research based on fractal dimension as feature and Adaboost as classifier. J. Neural Eng..

[32] Phothisonothai M., Nakagawa M. (2007). Fractal-Based EEG Data Analysis of Body Parts Movement imagery tasks. J. Physiol. Sci..

[33] Phothisonothai M., Nakagawa M. (2008). EEG-based classification of motor imagery tasks using fractal dimension and neural network for brain-computer interface. IEICE Trans. Inf. Syst..

[34] Güclü U., Güclütürk Y., Loo C.K. (2011). Evaluation of fractal dimension estimation methods for feature extraction in motor imagery based brain computer interface. Proced. Comput. Sci..

[35] Hsu W.Y. (2012). Fuzzy Hopfield neural network clustering for single-trial motor imagery EEG classification. Expert Syst. Appl..

[36] Aguilar J.M., Castillo J., Elias D. EEG signals processing based on fractal dimension features and classified by neural network and support vector machine in motor imagery for a BCI. Proceedings of the VI Latin American Congress on Biomedical Engineering CLAIB.

[37] Peng C.K., Mietus J., Hausdorff J.M., Havlin S., Stanley H.E., Goldberger A.L. (1993). Long-range anticorrelations and non-Gaussian behavior of the heartbeat. Phys. Rev. Lett..

[38] Katz M.J. (1988). Fractals and the analysis of waveforms. Comput. Biol. Med..

[39] Higuchi T. (1988). Approach to an irregular time series on the basis of the fractal theory. Phys. D Nonlinear Phenom..

[40] Hurst H.E. (1951). Long-term storage capacity of reservoirs. Am. Soc. Civil Eng..

[41] Grassberger P., Procaccia I. (1983). Measuring the strangeness of attractors. Phys. D Nonlinear Phenom..

[42] Wang S., Liu C.L., Zheng L. Feature selection by combing Fisher criterion and principal feature analysis. Proceedings of the International Joint Conference on Machine Learning and Cybernetics.

[43] Fang L., Zhao H., Wang P., Yu M., Yan J., Cheng W., Chen P. (2015). Feature selection method based on mutual information and class separability for dimension reduction in multidimensional time series for clinical data. Biomed. Signal Process. Control.

[44] Naseer N., Hong K.-S. (2013). Classification of functional near-infrared spectroscopy signals corresponding to right- and left-wrist motor imagery for development of a brain-computer interface. Neurosci. Lett..

[45] Guo L., Rivero D., Dorado J., Munteanu C.R., Pazos A. (2011). Automatic feature extraction using genetic programming: An application to epileptic EEG classification. Expert Syst. Appl..

[46] Naseer N., Noori F.M., Qureshi N.K., Hong K.-S. (2016). Determining optimal feature-combination for LDA classification of functional near-infrared spectroscopy signals in brain-computer interface application. Front. Hum. Neurosci..

[47] Cedarbaum J.M., Stambler N., Malta E., Fuller C., Hilt D., Thurmond B., Nakanishi A. (1999). The ALSFRS-R: A revised ALS functional rating scale that incorporates assessments of respiratory function. J. Neurol. Sci.

[48] Roche J.C., Rojas-Garcia R., Scott K.M., Scotton W., Ellis C.E., Burman R., Wijesekera L., Turner M.R., Leigh P.N., Shaw C.E. (2012). A proposed staging system for amyotrophic lateral sclerosis. Brain.

[49] Balendra R., Jones A., Jivraj N., Steen I.N., Young C.A., Shaw P.J., Turner M.R., Leigh P.N., Al-Chalabi A. (2015). Use of clinical staging in amyotrophic lateral sclerosis for phase 3 clinical trials. J. Neurol. Neurosurg. Psychiatry.

[50] Hsu W.C., Lin L.F., Chou C.W., Hsiao Y.T., Liu Y.H. (2017). EEG classification of imaginary lower limb stepping movements based on fuzzy support vector machine with kernel-induced membership function. Int. J. Fuzzy Syst..

[51] Esteller R., Vachtsevanos G., Echauz J. (2001). A comparison of waveform fractal dimension. IEEE. Trans. Circuits Syst..

[52] Ramoser H., Müller-Gerking J., Pfurtscheller G. (2000). Optimal spatial filtering of single trial EEG during imagined hand movement. IEEE Trans. Rehab. Eng..

[53] Tang Z., Sun S., Zhang S., Chen Y., Li C., Chen S. (2016). A Brain-Machine Interface Based on ERD/ERS for an Upper-Limb Exoskeleton Control. Sensors.

[54] Zhang Y., Liu B., Ji X., Huang D. (2017). Classification of EEG Signals Based on Autoregressive Model and Wavelet Packet Decomposition. Neural Process. Lett..

[55] Blankertz B., Tomioka R., Lemm S., Kawanabe M., Müller K.-R. (2008). Optimizing spatial filters for robust EEG single-trial analysis. IEEE Sign. Process. Mag..

[56] Cecotti H., Gräser A. (2011). Convolutional Neural Networks for P300 Detection with Application to Brain-Computer Interfaces. IEEE Trans. Pattern Anal. Mach. Intell..

[57] Qian K., Nikolov P., Huang D., Fei D.Y., Chen X., Bai O. (2010). A motor imagery-based online interactive brain-controlled switch: Paradigm development and preliminary test. Clin. Neurophysiol..

[58] Bennasar M., Hicks Y., Setchi R. (2015). Feature selection using joint mutual information maximization. Expert Syst. Appl..

[59] Gonzalez A., Nambu I., Hokari H., Wada Y. (2014). EEG channel selection using particle swarm optimization for the classification of auditory event-related potentials. Sci. World J..

